# MARSI: metabolite analogues for rational strain improvement

**DOI:** 10.1093/bioinformatics/bty108

**Published:** 2018-02-23

**Authors:** João G R Cardoso, Ahmad A Zeidan, Kristian Jensen, Nikolaus Sonnenschein, Ana Rute Neves, Markus J Herrgård

**Affiliations:** 1 The Novo Nordisk Foundation Center for Biosustainability, Technical University of Denmark, Kongens Lyngby, Denmark; 2 Discovery, Chr. Hansen A/S, Hørsholm, Denmark

## Abstract

**Summary:**

Metabolite analogues (MAs) mimic the structure of native metabolites, can competitively inhibit their utilization in enzymatic reactions, and are commonly used as selection tools for isolating desirable mutants of industrial microorganisms. Genome-scale metabolic models representing all biochemical reactions in an organism can be used to predict effects of MAs on cellular phenotypes. Here, we present the metabolite analogues for rational strain improvement (MARSI) framework. MARSI provides a rational approach to strain improvement by searching for metabolites as targets instead of genes or reactions. The designs found by MARSI can be implemented by supplying MAs in the culture media, enabling metabolic rewiring without the use of recombinant DNA technologies that cannot always be used due to regulations. To facilitate experimental implementation, MARSI provides tools to identify candidate MAs to a target metabolite from a database of known drugs and analogues.

**Availability and implementation:**

The code is freely available at https://github.com/biosustain/marsi under the Apache License V2. MARSI is implemented in Python.

**Supplementary information:**

Supplementary data are available at *Bioinformatics* online.

## 1 Introduction

Genome-scale metabolic models (GEMs) describe the biochemical reactions in an organism and their relation to the proteome and genome (McCloskey *et al.*, 2013). These models comprehensively represent natural metabolism and they are useful for predicting the effect of metabolite analogues (MAs) as therapeutics (Agren *et al.*, 2014; Kim *et al.*, 2014).

Non-rational strategies such as mutagenesis and selection or laboratory evolution can be used to develop industrial strains when the use of recombinant DNA technology is not allowed due to regulations (Derkx *et al.*, 2014; Hansen *et al.*, 2017). MAs, inhibiting the enzymatic conversion of the target metabolite, act as metabolite knockouts and can be used as the selective pressure in non-rational strategies to shape the metabolism of microorganisms (Sørensen *et al.*, 2016).

**Fig. 1.**
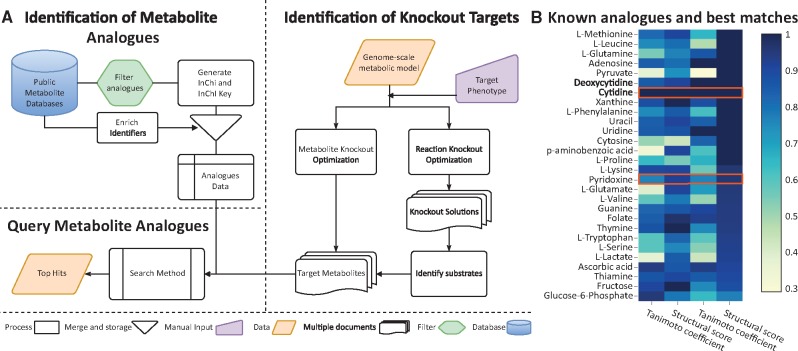
Metabolite target identification workflow and examples of MA targets. **(A)** The workflow for identifying for metabolite knockouts and candidate MAs. **(B)** Comparison between the known MAs (columns 1 and 2) and the best MARSI hits (columns 3 and 4) used to calibrate the search parameters. We show the TC and the SS. We highlighted rows where the best MARSI hit and the known MA are the same

Here, we present software that implements workflows to identify metabolite knockouts instead of gene or reaction knockouts (Figure 1A). We also provide a pipeline to identify structural analogues for those targets.

## 2 Materials and methods

The first workflow consists of systematically replacing reaction knockouts (identified by other strain design methods) by metabolite knockouts, until we can find metabolite targets that result in a similar flux distribution. The second workflow consists of searching for metabolite targets using heuristic optimization, without the need to specify reaction knockouts a priori. A metabolite knockout consists of blocking all reactions consuming a given metabolite, excluding transporters.

After identifying the metabolite targets, we search for MAs similar to them. We compiled a database of potential MAs from publicly available sources (see Supplementary Material). We use OpenBabel (O’Boyle *et al.*, 2011) and RDKit (2017) (http://www.rdkit.org) to calculate the features used to compare candidate MAs to the target metabolite: number of atoms/bonds/rings, MACCs fingerprints, Tanimoto coefficient (TC) and structural similarity score (SS).

## 3 Results

We implemented a software package containing algorithms to generate strain design strategies using MAs. Our software could generate metabolite targets for a published knockout-based design (Harder *et al.*, 2016). We also provide the tools to identify candidate MAs that could be used for implementation of the designs.

### 3.1 Identification of replacement targets

We used an experimentally validated strain design for itaconic acid production in *Escherichia coli* (Harder *et al.*, 2016) and the *E.coli* GEM iJO1366 (Orth *et al.*, 2014) to demonstrate the use of MARSI. MARSI identified acetyl-phosphate as a metabolite knockout target that can replace the Phosphotransacetylase (PTAr) reaction knockout and sustain the same flux for itaconic acid production (Table 1). Using a SS cut-off of 0.5 (see Supplementary Material), we found 182 MAs for acetyl-phosphate (Supplementary Table S1 shows the top 10 hits). More examples of replacement targets in other *E.coli* strain designs can be found in Supplementary Material.

**Table 1.** Knockout replacements for the strain design

Non-replaced knockouts	Replaced reaction	Metabolite	Original fitness	New fitness
PTA2, ICL, ALDD2x, PYK, SUCOAS, GGGABADr	PTAr	Acetyl-P	0.001	0.001

We use Biomass Product Coupled Yield (Patil *et al.*, 2005) as fitness measure. Reaction Ids: Phosphate acetyltransferase (PTA), Isocitrate lyase (ICL), Aldehyde dehydrogenase (ALDD2x), Pyruvate kinase (PK), Succinyl-CoA synthetase (SUCOAS) and Gamma-glutamyl-gamma aminobutyric acid dehydrogenase (GGGABADr).

### 3.2 Query calibration with known MAs

In order to validate the ability of MARSI to find known analogues for a target metabolite, we selected 42 known metabolite-MA pairs from the literature (Supplementary Table S3). We compared the structural features between the MAs and their target metabolites (Supplementary Fig. S1). We used a distance of 4 for the number of atoms, 3 for the number of bonds and 2 for the number of rings as our query cut-off. The TC cut-off changes dynamically with the size of the metabolites (see Supplementary Material). In Figure 1B, we show the SS and TC for different targets and their known analogues as well as the best hit analogue in the database. For most targets MARSI found candidate MAs that showed higher structural similarity to the target metabolite than the known analogue.

## Supplementary Material

Supplementary Data
