# Supplementary material for: MARSI: metabolite analogues for rational strain improvement
Source: Bioinformatics. 2018 Feb 23;34(13):2319–21. doi: 10.1093/bioinformatics/bty108 (PMC6022549; doi:10.1093/bioinformatics/bty108)
Supplement: Supplementary Data [file bty108_supplementary_materials_revision.docx]

MARSI: metabolite analogues for rational strain improvement

João G. R. Cardoso^1^, Ahmad A. Zeidan^2,*^, Kristian Jensen^1^, Nikolaus Sonnenschein^1^, Ana Rute Neves^2^ and Markus J. Herrgård^1, *^

^1^The Novo Nordisk Foundation Center for Biosustainability, Technical University of Denmark, Denmark

^2^Discovery, Chr. Hansen A/S, DK-2970, Hørsholm, Denmark

* To whom correspondence should be addressed

**Metabolite knockouts**

To knockout a metabolite we block all reactions consuming a given metabolite in all compartments. Because we assume the use of metabolite analogues, we ignore transport reactions. We use a reference flux distribution to identify which reactions consume a metabolite. If no reference is available, we block all reactions consuming that metabolite.

**Metabolite analogues database**

We compiled a comprehensive chemical compound database using publicly available data (see Table S2). We also included some known analogues retrieved from the literature (Table S3).

**Structural score**

Given two compounds X and Y, we can determine the maximum common substructure (MCS) Z using the RDKit API.

Let Z be $\boldsymbol{MCS}(\boldsymbol{X}, \boldsymbol{Y})$, the similarity between X and Y is given by the function S as follows:

$$\boldsymbol{S}\left( \boldsymbol{X}, \boldsymbol{Y, \alpha, \beta} \right)=\boldsymbol{s}\left( \boldsymbol{X}, \boldsymbol{Z, \alpha, \beta} \right)\cdot\boldsymbol{s}\left( \boldsymbol{Y}, \boldsymbol{Z, \alpha, \beta} \right)$$

where *s* is the similarity between a molecule and Z. The function *s* is defined the following way:

$$s\left( mol, mcs \right)=\alpha\boldsymbol{\cdot}\frac{\boldsymbol{mcs}\boldsymbol{.}\boldsymbol{atoms}}{\boldsymbol{mol}.\boldsymbol{atoms}} + \beta\cdot\frac{\boldsymbol{mcs}.\boldsymbol{bonds}}{\boldsymbol{mol}.\boldsymbol{bonds}}$$

The atoms and bonds in Z is always lower or equal to either molecule X or Y. If $\boldsymbol{alp}\mathbf{h}\boldsymbol{a} + \boldsymbol{beta} = \mathbf{1}$, the similarity (*S*) is always [0, 1] The MCS algorithm provided by RDKit needs some parameterization. Because we want things that have a similar structure regardless of the atom substitutions we run the algorithm using the following parameters: maximize bounds, ignore atoms types and bond types but still forcing the rings to match.

With a structural similarity cutoff of 0.5, we can capture most of the known metabolite analogues (Figure S2).

**Dynamic Tanimoto coefficient cutoff**

Small metabolites share a lower Tanimoto coefficient with their known analogues (Figure S3). To ensure that we can also reach those metabolites, we use a dynamic coefficient based on the following equation:

$$T_{cutoff}= min(0.75, 0.017974*mol.atoms + 0.008239)$$

The slope of the line was calculated using the linear least-squares regression for between the number of atoms, *#atoms*, and the Tanimoto coefficients for all known metabolite analogues. We adjusted the intercept to include all known analogues by subtracting 0.4 to the computed value.

**Database Query**

The database query is done in three steps: filter metabolites by size (i.e., number of atoms, number of rings and number of bonds), filter metabolites by Tanimoto coefficient, and sort by structural score. In the first step, we retrieve all entries (*y*) from the database that match the query (*x*) using the following criteria:

$$x.atoms-4\leq y.atoms\leq x.atoms+4$$

$$x.bonds-3\leq y.bonds\leq x.bonds+3$$

$$x.rings-2\leq y.rings\leq x.rings+2$$

Then we compute the Tanimoto coefficient for all the retrieved entries and we keep the ones above the cutoff. Finally, we compute the structural score for the remaining entries and sort them in descending order using the structural score. Table S6 shows that we can find metabolite analogues for different metabolites present in the *E. coli* model.

**Heuristic optimization search (OptMet)**

We implemented a search method that identifies metabolite targets without the need to predict reaction knockouts. To do that, we use evolutionary algorithms. These algorithms use heuristic approaches inspired in Darwinian evolution to optimize problems with high combinatorial complexity. They do not always guarantee the optimum result, but the solutions found are very close to the global optimum and they have a much lower computational cost. In a simple evolutionary algorithm, there is a representation of the possible states, called the genome (Patil *et al.*, 2005).

The OptMet algorithm (described in figure S4) works as follows:

1. Initialize a random population. Each individual, (i.e., a member of the population) is represented as a genome vector and it corresponds to a possible solution. The genome vector, has *n* elements and the value of each element is True or False (or equivalent 1 or 0). In this case, the *n* is the number of non-essential metabolites.
2. Evaluate the population. Each individual (a possible solution in the population) is evaluated using flux balance analysis (or an equivalent function). In each evaluation, if the *i*th position of the vector is True, then we block the usage of a given metabolite. The fitness of each individual is calculated using the flux distribution, given an objective function. We store the best result of every iteration.
3. If any termination criteria are met (e.g., the maximum number of iterations, a time limit, etc.) then return the best individual. Otherwise continue.
4. Apply the operators. The operators change the population. They select the best individuals (selection), create the progeny (cross-over) and change the new individuals (mutation). A new population is created.
5. Go to step 2.

**Additional replacement designs**

We selected a subset of published growth-coupled designs for *Escherichia* *coli* that can be reproduced using the latest *E. coli* GEM (Table S4) (King *et al.*, 2016). Then, we used our algorithm to identify for metabolite knockout targets that could be used to replace one reaction knockout for each design (Table S5). We identified 11 different metabolites that can be used as replacements targets. Computational implementation of the original designs and fitness calculations were done using *cameo*, a python library for computer-aided metabolic engineering and optimization (Cardoso *et al.*, 2017).

**Metabolite analogs available for *E. coli***

We used MARSI to identify possible analogs for metabolites native in *E. coli.* From the X non-essential metabolites, we found candidate analogs in our database for Y. Figure S5 summarizes the distribution of metabolites available for *E. coli* metabolism with a structural similarity score above 0.5.

References

Cardoso,J.G.R. *et al.* (2017) Cameo : A Python Library for Computer Aided Metabolic Engineering and Optimization of Cell Factories. *bioRxiv*.

Irwin,J.J. *et al.* (2012) ZINC: A free tool to discover chemistry for biology. *J. Chem. Inf. Model.*, **52**, 1757–1768.

Kanehisa,M. *et al.* (2017) KEGG: new perspectives on genomes, pathways, diseases and drugs. *Nucleic Acids Res.*, **45**, 353–61.

Kim,S. *et al.* (2016) PubChem substance and compound databases. *Nucleic Acids Res.*, **44**, D1202–D1213.

King,Z.A. *et al.* (2016) Literature mining supports a next-generation modeling approach to predict cellular byproduct secretion. *Metab. Eng.*, 1–8.

Matos,P. de *et al.* (2010) Chemical Entities of Biological Interest: an update. *Nucleic Acids Res.*, **38**, D249--54.

Patil,K.R. *et al.* (2005) Evolutionary programming as a platform for in silico metabolic engineering. *BMC Bioinformatics*, **6**, 308.

Wishart,D.S. *et al.* (2006) DrugBank: a comprehensive resource for in silico drug discovery and exploration. *Nucleic Acids Res.*, **34**, D668–D672.

Table S1 Top 10 analogue matches for acetyl-phosphate

Table S2 Chemical Databases and queries to retrieve the chemical compounds found in the analogues database.

| **Database** | **Query/File** | **Comments** |
| --- | --- | --- |
| KEGG (Kanehisa et al., 2017) | BR08310 | Enzyme inhibitors |
| ChEBI (Matos et al., 2010) | CHEBI:35221 | ‘has role’ or ‘is a’ ontology children |
| PubChem Compound (Kim et al., 2016) | (antimetabolites) OR (analog) OR (analogue) | Retrieved the summary as file |
| DrugBank (Wishart et al., 2006) | All open structures |  |
| ZINC (Irwin et al., 2012) | ‘All clean’ dataset | From ZINC12 |

Table S3 List of known metabolite analogues manually collected from databases and literature.

| **Database** | **Identifier** | **Name** | **Target** | **Target Identifier** | **PMID** | **Group** |
| --- | --- | --- | --- | --- | --- | --- |
| PubChem | 89034 | Methionine sulphoximine | L-Glutamate | CHEBI:29985 |  | Amino acids |
| ChEBI | CHEBI:74545 | acivicin | L-Glutamine | CHEBI:18050 |  | Amino acids |
| ChEBI | CHEBI:85005 | anticapsin | L-Glutamine | CHEBI:18050 |  | Amino acids |
| ChEBI | CHEBI:74846 | azaserine | L-Glutamine | CHEBI:18050 |  | Amino acids |
| ChEBI | CHEBI:18347 | L-norleucine | L-Leucine | CHEBI:15603 |  | Amino acids |
| ChEBI | CHEBI:42101 | D-norleucine | L-Leucine | CHEBI:15603 |  | Amino acids |
| PubChem | 3032849 | 5-hydroxylysine | L-Lysine | CHEBI:18019 | 6806159 | Amino acids |
| ChEBI | CHEBI:497734 | thialysine | L-Lysine | CHEBI:18019 | 6806159 | Amino acids |
| PubChem | 99558 | S-2-aminoethyl-L- cysteine | L-Lysine | CHEBI:18019 | 1841850 | Amino acids |
| ChEBI | CHEBI:4886 | L-ethionine | L-Methionine | CHEBI:16643 |  | Amino acids |
| PubChem | 146719 | Thienylalanine | L-Phenylalanine | CHEBI:17295 |  | Amino acids |
| PubChem | 16486 | Azetidine carboxylic acid | L-Proline | CHEBI:17203 |  | Amino acids |
| ChEBI | CHEBI:75494 | serine hydroxamate | L-Serine | CHEBI:17115 | PMC248741 | Amino acids |
| ChEBI | CHEBI:72341 | O-(2-aminoethyl)-L-serine | L-Serine | CHEBI:17115 |  | Amino acids |
| PubChem | 150990 | 5-Methyl-DL-tryptophan | L-Tryptophan | CHEBI:16828 |  | Amino acids |
| PubChem | 9577 | DL-5-Fluorotryptophan | L-Tryptophan | CHEBI:16828 | PMC429912 | Amino acids |
| ChEBI | CHEBI:18314 | L-Norvaline | L-Valine | CHEBI:16414 |  | Amino acids |
| ChEBI | CHEBI:28804 | D-Norvaline | L-Valine | CHEBI:16414 |  | Amino acids |
| PubChem | 5790 | Floxuridine | Uridine | CHEBI:16704 | PMC2827868 | Nucleotides |
| PubChem | 119182 | Clofarabine | Adenosine | CHEBI:16335 | PMC2827868 | Nucleotides |
| PubChem | 439693 | Pentostatin | Adenosine | CHEBI:16335 | PMC2827868 | Nucleotides |
| PubChem | 3011155 | Nelarabine | Adenosine | CHEBI:16335 | PMC2827868 | Nucleotides |
| PubChem | 20279 | Cladribine | Adenosine | CHEBI:16335 | PMC2827868 | Nucleotides |
| PubChem | 451668 | Decitabine | Cytidine | CHEBI:17562 | PMC2827868 | Nucleotides |
| PubChem | 20279 | Cladribine | Cytidine | CHEBI:17562 | PMC2827868 | Nucleotides |
| PubChem | 6253 | Cytarabine | Cytidine | CHEBI:17562 | PMC2827868 | Nucleotides |
| PubChem | 9444 | Vidaza | Cytosine | CHEBI:16040 | PMC2827868 | Nucleotides |
| PubChem | 60750 | Gemcitabine | Deoxycytidine | CHEBI:15698 | PMC2827868 | Nucleotides |
| PubChem | 119182 | Clofarabine | Guanine | CHEBI:16235 | PMC2827868 | Nucleotides |
| PubChem | 2723601 | 6-thioguanine | Guanine | CHEBI:16235 | PMC2827868 | Nucleotides |
| PubChem | 9444 | Vidaza | Thymine | CHEBI:17821 | PMC2827868 | Nucleotides |
| PubChem | 5802 | Bromouracil | Thymine | CHEBI:17821 |  | Nucleotides |
| PubChem | 3385 | 5-fluorouracil | Uracil | CHEBI:17568 | PMC2827868 | Nucleotides |
| ChEBI | CHEBI:28315 | alloxanthine | Xanthine | CHEBI:17712 |  | Nucleotides |
| PubChem | 8646 | 8-Azaguanine | Guanine | CHEBI:16235 |  | Nucleotides |
| PubChem | 974 | Oxamate | L-Lactate | CHEBI:16651 |  | Organic Acids |
| ChEBI | CHEBI:45373 | sulfanilamide | p-aminobenzoic acid | CHEBI:30753 | PMC3361698 | Organic Acids |
| PubChem | 656481 | methyl-acetylphosphonate | Pyruvate | CHEBI:15361 |  | Organic Acids |
| PubChem | 73544 | 2,5-Anhydro-D-mannito | Fructose | CHEBI:28645 | 8374733 | Sugars |
| PubChem | 440992 | 2-Deoxy-Glucose-6P | Glucose-6-Phosphate | CHEBI:17665 |  | Sugars |
| PubChem | 54679283 | glucoascorbic acid | Ascorbic acid | CHEBI:29073 |  | Vitamins |
| PubChem | 80058 | triethylcholine | Choline | CHEBI:15354 |  | Vitamins |
| PubChem | 169371 | Aminopterin | Folate | CHEBI:62501 |  | Vitamins |
| PubChem | 6094 | 4-deoxypyridoxine | Pyridoxine | CHEBI:16709 |  | Vitamins |
| ChEBI | CHEBI:72290 | pyrithiamine | Thiamine | CHEBI:18385 |  | Vitamins |

Table S4 Strain designs validated experimentally and reproduced in silico (King et al., 2016). If the “fva min” value is greater than 0, then the design is growth coupled. “+reaction” means that a heterologous reaction was inserted and “-reaction” means that a reaction was knocked out. The “target” refers to exchange reaction for the target product that is produced in the design. The “substrate” column refers to the media composition (assuming a base M9 minimal medium).

| **Key** | **Target** | **Substrate** | **Aerobicity** | **Growh rate** | **Fva min** | **Strain design** |
| --- | --- | --- | --- | --- | --- | --- |
| A1 | EX_etoh_e | glucose+LB | anaerobic | 0,142 | 4,8695 | +reaction.PDC |
| B1 | EX_lac__D_e | glucose | anaerobic | 0,1948 | 16,8641 | -reaction.ACALD, -reaction.ALCD2x,  -reaction.PTA2, -reaction.PTAr |
| C1 | EX_etoh_e | glucose+LB | anaerobic | 0,105 | 0 | -reaction.FRD2, -reaction.FRD3, -reaction.OBTFL, +reaction.PDC, -reaction.PFL |
| D1 | EX_succ_e | glucose+LB | microaerobic | 0,1051 | 0,0329 | -reaction.LDH_D, -reaction.OBTFL, -reaction.PFL |
| E1 | EX_succ_e | glucose | anaerobic | 0,1892 | 0,0627 | -reaction.LDH_D, -reaction.OBTFL, -reaction.PFL |
| F1 | EX_succ_e | glucose | anaerobic | 0,1611 | 0,0534 | -reaction.ACGAptspp, -reaction.GLCptspp,  -reaction.LDH_D, -reaction.OBTFL, -reaction.PFL |
| G1 | EX_lac__L_e | glucose | anaerobic | 0,1972 | 0 | -reaction.LDH_D, +reaction.LDH_L,  -reaction.PTA2, -reaction.PTAr |
| H1 | EX_lac__D_e | glucose | anaerobic | 0,1388 | 0 | -reaction.PPC, -reaction.PTA2, -reaction.PTAr |
| I1 | EX_lac__L_e | xylose | anaerobic | 0,1237 | 0 | -reaction.LDH_D, +reaction.LDH_L,  -reaction.OBTFL, -reaction.PFL |
| A2 | EX_lac__L_e | glucose | anaerobic | 0,1892 | 0 | -reaction.LDH_D, +reaction.LDH_L,  -reaction.OBTFL, -reaction.PFL |
| B2 | EX_succ_e | glucose+LB | anaerobic | 0,0769 | 0,0255 | -reaction.ACGAptspp, -reaction.GLCptspp,  -reaction.LDH_D, -reaction.OBTFL, -reaction.PFL, +reaction.PYC |
| C2 | EX_pyr_e | glucose+acetate | microaerobic | 0,1063 | 0 | -reaction.LDH_D, -reaction.OBTFL, -reaction.PDH,  -reaction.PFL, -reaction.POX, -reaction.PPS |
| D2 | EX_lac__L_e | glucose | anaerobic | 0,1878 | 16,7322 | -reaction.ACALD, -reaction.ACKr,  -reaction.ALCD2x, -reaction.FORt2pp,  -reaction.FORtppi, -reaction.FRD2, -reaction.FRD3,  -reaction.LDH_D, +reaction.LDH_L,  -reaction.OBTFL, -reaction.PFL |
| E2 | EX_lac__D_e | glucose | anaerobic | 0,1878 | 16,7322 | -reaction.ACALD, -reaction.ACKr,  -reaction.ALCD2x, -reaction.FORt2pp,  -reaction.FORtppi, -reaction.FRD2, -reaction.FRD3,  -reaction.OBTFL, -reaction.PFL |
| F2 | EX_ac_e | glucose | aerobic | 0,4025 | 12,7299 | -reaction.ACALD, -reaction.AKGDH,  -reaction.ALCD2x, -reaction.ATPS4rpp,  -reaction.FORt2pp, -reaction.FORtppi,  -reaction.FRD2, -reaction.FRD3, -reaction.LDH_D,  -reaction.OBTFL, -reaction.PFL |
| G2 | EX_pyr_e | glucose+acetate | microaerobic | 0,1063 | 0 | -reaction.LDH_D, -reaction.OBTFL, -reaction.PDH,  -reaction.PFL, -reaction.POX, -reaction.PPS |
| H2 | EX_lac__D_e | glucose | anaerobic | 0,1948 | 16,8641 | -reaction.ACALD, -reaction.ALCD2x,  -reaction.PTA2, -reaction.PTAr |
| I2 | EX_lac__D_e | glucose | anaerobic | 0,193 | 0 | -reaction.PFK, -reaction.PFK_2, -reaction.PFK_3,  -reaction.PTA2, -reaction.PTAr |
| A3 | EX_lac__D_e | glucose | anaerobic | 0,1908 | 16,929 | -reaction.ACALD, -reaction.ALCD2x,  -reaction.HEX1, -reaction.PFK, -reaction.PFK_2,  -reaction.PFK_3, -reaction.PTA2, -reaction.PTAr |
| B3 | EX_succ_e | glucose+LB | microaerobic | 0 | 0 | -reaction.ACKr, -reaction.ICDHyr, -reaction.POX,  -reaction.PTA2, -reaction.PTAr, -reaction.SUCDi |
| C3 | EX_succ_e | glucose+LB | anaerobic | 0,085 | 0,0282 | -reaction.ACALD, -reaction.ACGAptspp,  -reaction.ALCD2x, -reaction.GLCptspp,  -reaction.LDH_D, +reaction.PYC |
| D3 | EX_succ_e | pyruvate+yeast extract | anaerobic | 0 | 0 | -reaction.ACGAptspp, -reaction.GLCptspp,  -reaction.PYK |
| E3 | EX_succ_e | glucose+LB | microaerobic | 0 | 0 | -reaction.ACKr, -reaction.ICDHyr, -reaction.POX,  -reaction.PTA2, -reaction.PTAr, -reaction.SUCDi |
| F3 | EX_succ_e | glucose | anaerobic | 0,1639 | 0,0543 | -reaction.ACALD, -reaction.ACKr,  -reaction.ALCD2x, -reaction.LDH_D,  -reaction.PTA2, -reaction.PTAr, +reaction.PYC |
| G3 | EX_succ_e | glucose+yeast extract | anaerobic | 0,0674 | 0,0223 | -reaction.ACGAptspp, -reaction.GLCptspp,  -reaction.PYK |
| H3 | EX_lac__D_e | glucose | anaerobic | 0,1878 | 16,7322 | -reaction.ACALD, -reaction.ACKr,  -reaction.ALCD2x, -reaction.FRD2, -reaction.FRD3,  -reaction.OBTFL, -reaction.PFL |
| I3 | EX_lac__L_e | glucose | anaerobic | 0,1872 | 17,2334 | -reaction.ACALD, -reaction.ACKr,  -reaction.ALCD2x, -reaction.FRD2, -reaction.FRD3, -reaction.LDH_D, +reaction.LDH_L,  -reaction.MGSA, -reaction.OBTFL, -reaction.PFL |
| A4 | EX_lac__D_e | glucose+betaine | anaerobic | 0,1878 | 16,7322 | -reaction.ACALD, -reaction.ACKr,  -reaction.ALCD2x, -reaction.FRD2, -reaction.FRD3,  -reaction.OBTFL, -reaction.PFL |
| B4 | EX_lac__D_e | glucose | anaerobic | 0,1872 | 17,2334 | -reaction.ACALD, -reaction.ACKr,  -reaction.ALCD2x, -reaction.FRD2, -reaction.FRD3, -reaction.MGSA, -reaction.OBTFL, -reaction.PFL |
| C4 | EX_ala__L_e | glucose+betaine | anaerobic | 0,0829 | 0 | -reaction.ACALD, -reaction.ACKr, +reaction.ALADH_L, -reaction.ALCD2x,  -reaction.FRD2, -reaction.FRD3, -reaction.LDH_D,  -reaction.MGSA, -reaction.OBTFL, -reaction.PFL |
| D4 | EX_ala__L_e | glucose+betaine | anaerobic | 0 | 0 | -reaction.ACALD, -reaction.ACKr, +reaction.ALADH_L, -reaction.ALAR,  -reaction.ALCD2x, -reaction.FRD2, -reaction.FRD3,  -reaction.LDH_D, -reaction.MGSA, -reaction.OBTFL, -reaction.PFL |
| E4 | EX_h2_e | glucose | anaerobic | 0,2878 | 16,7623 | -reaction.FDH4pp, -reaction.FDH5pp,  -reaction.FRD2, -reaction.FRD3, -reaction.HYD1pp,  -reaction.HYD2pp, -reaction.HYD3pp,  -reaction.LDH_D, +reaction.PDC, -reaction.PDH |
| F4 | EX_lac__D_e | glucose | anaerobic | 0,1889 | 17,2079 | -reaction.FRD2, -reaction.FRD3, -reaction.OBTFL,  -reaction.PDH, -reaction.PFL, -reaction.POX,  -reaction.PPS |
| G4 | EX_etoh_e | glycerol | anaerobic | 0,0541 | 0,7119 | -reaction.FRD2, -reaction.FRD3, -reaction.PTA2,  -reaction.PTAr |
| H4 | EX_1poh_e | D-Glucose+L-Valine+L-Isoleucine+L-Leucine | microaerobic | 0 | 0 | +reaction.1PDH, +reaction.2OBUTDC,  -reaction.ACALD, -reaction.ALCD2x,  -reaction.DHAD1, -reaction.DHAD2, +reaction.EX_1poh_e, +reaction.EX_2mbtoh_e, +reaction.EX_2phetoh_e, +reaction.EX_iamoh_e,  -reaction.FRD2, -reaction.FRD3, -reaction.LDH_D,  -reaction.PTA2, -reaction.PTAr |
| I4 | EX_iboh_e | glucose | microaerobic | 0,164 | 0 | +reaction.3MOBDC, -reaction.ACALD,  -reaction.ALCD2x, +reaction.EX_2mbtoh_e, +reaction.EX_2phetoh_e, +reaction.EX_iamoh_e, +reaction.EX_iboh_e, -reaction.FRD2,  -reaction.FRD3, +reaction.IBDH, -reaction.LDH_D,  -reaction.PTA2, -reaction.PTAr |
| A5 | EX_1boh_e | D-Glucose+L-Valine+L-Isoleucine+L-Leucine | microaerobic | 0 | 0 | +reaction.1BDH, +reaction.2KVDC,  -reaction.ACALD, -reaction.ALCD2x,  -reaction.DHAD1, -reaction.DHAD2, +reaction.EX_1boh_e, +reaction.EX_2phetoh_e,  -reaction.FRD2, -reaction.FRD3, +reaction.ILV_PATHWAY, -reaction.LDH_D,  -reaction.PTA2, -reaction.PTAr |
| B5 | EX_etoh_e | glycerol | anaerobic | 0,0541 | 0,7119 | -reaction.FHL, -reaction.FRD2, -reaction.FRD3,  -reaction.PTA2, -reaction.PTAr |
| C5 | EX_etoh_e | glucose+LB | anaerobic | 0,0792 | 5,3695 | -reaction.ACGAptspp, -reaction.ACMANAptspp,  -reaction.FRD2, -reaction.FRD3,  -reaction.FRUpts2pp, -reaction.G6PDH2r,  -reaction.GAMptspp, -reaction.GLCptspp,  -reaction.HEX1, -reaction.LDH_D,  -reaction.MANptspp, -reaction.ME2,  -reaction.NADH10, -reaction.NADH5,  -reaction.NADH9, -reaction.POX, -reaction.PTA2,  -reaction.PTAr |
| D5 | EX_iamoh_e | glucose | anaerobic | 0 | 0 | +reaction.3M1BDH, +reaction.4MOBDC,  -reaction.ACALD, -reaction.ALCD2x, +reaction.EX_iamoh_e, -reaction.FRD2,  -reaction.FRD3, -reaction.ILETA, -reaction.LDH_D, -reaction.LEUTAi, -reaction.OBTFL, -reaction.PFL,  -reaction.PHETA1, -reaction.PTA2, -reaction.PTAr,  -reaction.TYRTA, -reaction.VALTA |
| E5 | EX_succ_e | glucose | anaerobic | 0 | 0 | -reaction.ACALD, -reaction.ACKr,  -reaction.ALCD2x, -reaction.ASPTA,  -reaction.CITL, -reaction.FORt2pp,  -reaction.FORtppi, -reaction.LDH_D,  -reaction.MGSA, -reaction.OBTFL, -reaction.PFL,  -reaction.PHETA1, -reaction.POX, -reaction.PTA2,  -reaction.PTAr, -reaction.TYRTA |
| F5 | EX_1boh_e | glucose | microaerobic | 0,1972 | 8,2255 | +reaction.1BDH, -reaction.ACALD,  -reaction.ALCD2x, +reaction.B2COAR, +reaction.BTALDH, +reaction.EX_1boh_e,  -reaction.FRD2, -reaction.FRD3, -reaction.LDH_D,  -reaction.PTA2, -reaction.PTAr |
| G5 | EX_succ_e | glucose | anaerobic | 0 | 0 | -reaction.ACALD, -reaction.ACKr,  -reaction.ALCD2x, -reaction.ASPTA,  -reaction.CITL, -reaction.FORt2pp,  -reaction.FORtppi, -reaction.LDH_D,  -reaction.MGSA, -reaction.OBTFL, -reaction.PFL,  -reaction.PHETA1, -reaction.POX, -reaction.PTA2,  -reaction.PTAr, -reaction.TYRTA |
| H5 | EX_lac__D_e | glucose | aerobic | 0,8471 | 0 | -reaction.CYTBDpp, -reaction.CYTBO3_4pp,  -reaction.QMO2, -reaction.QMO3 |
| I5 | EX_succ_e | glucose+betaine | anaerobic | 0,1081 | 7,673 | -reaction.ACALD, -reaction.ACKr,  -reaction.ALCD2x, -reaction.FORt2pp,  -reaction.FORtppi, -reaction.LDH_D,  -reaction.MGSA, -reaction.OBTFL, -reaction.PFL,  -reaction.POX |
| A6 | EX_btd__RR_e | glucose+yeast extract | microaerobic | 0,0502 | 4,851 | -reaction.ACALD, +reaction.ACLDC,  -reaction.ALCD2x, +reaction.EX_btd__RR_e,  -reaction.FRD2, -reaction.FRD3, -reaction.LDH_D,  -reaction.OBTFL, -reaction.PFL, -reaction.PTA2,  -reaction.PTAr, +reaction.sADHx, +reaction.sADHy |
| B6 | EX_lac__D_e | glucose | anaerobic | 0,1878 | 16,7322 | -reaction.ACALD, -reaction.ALCD2x,  -reaction.FRD2, -reaction.FRD3, -reaction.OBTFL,  -reaction.PFL |
| C6 | EX_btd__meso_e | glucose+yeast extract | microaerobic | 0,0502 | 4,851 | -reaction.ACALD, +reaction.ACLDC,  -reaction.ALCD2x, +reaction.EX_btd__meso_e,  -reaction.FRD2, -reaction.FRD3, -reaction.LDH_D,  -reaction.OBTFL, -reaction.PFL, -reaction.PTA2,  -reaction.PTAr, +reaction.sADHx |
| D6 | EX_succ_e | glycerol | anaerobic | 0,0387 | 0,0128 | -reaction.ACALD, -reaction.ALCD2x,  -reaction.LDH_D, -reaction.POX, -reaction.PPC,  -reaction.PTA2, -reaction.PTAr, +reaction.PYC |
| E6 | EX_3hb_e | glucose | anaerobic | 0,1639 | 0 | +reaction.3HB_POLYM, -reaction.ACALD,  -reaction.ACKr, -reaction.ALCD2x, +reaction.EX_3hb_e, +reaction.EX_3hv_e,  -reaction.LDH_D, +reaction.PHPB, -reaction.POX,  -reaction.PTA2, -reaction.PTAr |
| F6 | EX_h2_e | glycerol | anaerobic | 0,1264 | 16,9207 | -reaction.FRD2, -reaction.FRD3 |
| G6 | EX_succ_e | glycerol | anaerobic | 0 | 0 | -reaction.ACGAptspp, -reaction.ACMANAptspp,  -reaction.ACMUMptspp, -reaction.ARBTptspp,  -reaction.ASCBptspp, -reaction.CHTBSptspp,  -reaction.DHAPT, -reaction.FRUpts2pp,  -reaction.FRUptspp, -reaction.GALTptspp,  -reaction.GAMptspp, -reaction.GLCptspp,  -reaction.MALTptspp, -reaction.MANGLYCptspp,  -reaction.MANptspp, -reaction.MNLptspp,  -reaction.OBTFL, -reaction.PFL, -reaction.SBTptspp, -reaction.SUCptspp, -reaction.TREptspp |
| H6 | EX_lac__D_e | glucose | microaerobic | 0,1273 | 10,0258 | -reaction.ACALD, -reaction.ACKr,  -reaction.ALCD2x, -reaction.PPC |
| I6 | EX_3hb_e | glucose | anaerobic | 0,1972 | 0 | +reaction.3HB_POLYM, -reaction.ACKr, +reaction.EX_3hb_e, +reaction.EX_3hv_e,  -reaction.LDH_D, +reaction.PHPB, -reaction.POX,  -reaction.PTA2, -reaction.PTAr |
| A7 | EX_3hb_co_la_e | glucose | microaerobic | 0,1273 | 0 | +reaction.3HB_LA_POLYM, -reaction.ACALD,  -reaction.ACKr, -reaction.ALCD2x, +reaction.EX_3hb_co_la_e, +reaction.PHPB,  -reaction.PPC |
| B7 | EX_iboh_e | glucose+yeast extract | anaerobic | 0,0715 | 5,8784 | +reaction.3MOBDC, -reaction.ACALD,  -reaction.ALCD2x, +reaction.EX_2mbtoh_e, +reaction.EX_2phetoh_e, +reaction.EX_iamoh_e, +reaction.EX_iboh_e, -reaction.FRD2,  -reaction.FRD3, +reaction.IBDH, -reaction.LDH_D,  -reaction.OBTFL, -reaction.PFL, -reaction.PTA2,  -reaction.PTAr |
| C7 | EX_lac__D_e | glycerol | anaerobic | 0,0387 | 0 | -reaction.ACALD, -reaction.ALCD2x,  -reaction.FRD2, -reaction.FRD3, -reaction.LDH_D,  -reaction.LDH_D2, -reaction.PTA2, -reaction.PTAr |
| D7 | EX_3hb_e | glucose | anaerobic | 0,1081 | 0 | +reaction.3HB_POLYM, -reaction.ACALD,  -reaction.ACKr, -reaction.ALCD2x, +reaction.EX_3hb_e, +reaction.EX_3hv_e,  -reaction.LDH_D, -reaction.OBTFL, -reaction.PFL, +reaction.PHPB, -reaction.POX, -reaction.PTA2,  -reaction.PTAr |
| E7 | EX_14btd_e | glucose | microaerobic | 0,1791 | 5,0499 | +reaction.4HBACT, +reaction.4HBTALDDH,  -reaction.ACALD, +reaction.AKGDC,  -reaction.ALCD2x, +reaction.BTDP2, +reaction.EX_14btd_e, +reaction.EX_4hdxbld_e, +reaction.EX_gbl_e, +reaction.GBL_PROD,  -reaction.LDH_D, -reaction.MDH, -reaction.OBTFL, -reaction.PFL, +reaction.SUCCALDH |
| F7 | EX_iboh_e | glucose+yeast extract | anaerobic | 0,1077 | 0 | +reaction.3MOBDC, +reaction.EX_2mbtoh_e, +reaction.EX_2phetoh_e, +reaction.EX_iamoh_e, +reaction.EX_iboh_e, -reaction.FRD2,  -reaction.FRD3, -reaction.G6PDH2r, +reaction.IBDH, -reaction.LDH_D, -reaction.MDH,  -reaction.NADH10, -reaction.NADH5,  -reaction.NADH9, -reaction.POX, -reaction.PTA2,  -reaction.PTAr |
| G7 | EX_1boh_e | glucose+yeast extract | anaerobic | 0,1181 | 4,6922 | +reaction.1BDH, -reaction.ACALD,  -reaction.ALCD2x, +reaction.B2COAR, +reaction.BTALDH, +reaction.EX_1boh_e,  -reaction.FRD2, -reaction.FRD3, -reaction.LDH_D,  -reaction.PTA2, -reaction.PTAr |
| H7 | EX_1boh_e | glucose | anaerobic | 0,1972 | 1,2106 | +reaction.1BDH, -reaction.ACALD,  -reaction.ALCD2x, +reaction.B2COAR, +reaction.BTALDH, +reaction.EX_1boh_e,  -reaction.FRD2, -reaction.FRD3, -reaction.PTA2,  -reaction.PTAr |
| I7 | EX_succ_e | LB+glucose, sorbitol, and gluconate | microaerobic | 0,0303 | 8,404 | -reaction.ACALD, -reaction.ACGAptspp,  -reaction.ALCD2x, -reaction.GLCptspp,  -reaction.LDH_D, -reaction.OBTFL, -reaction.PFL |
| A8 | EX_lac__D_e | glucose | microaerobic | 0,1879 | 16,7322 | -reaction.ACALD, -reaction.ALCD2x,  -reaction.FRD2, -reaction.FRD3, -reaction.OBTFL,  -reaction.PFL |
| B8 | EX_1hex_e | glucose+yeast extract | anaerobic | 0,1181 | 3,1281 | +reaction.1HDH, -reaction.ACALD,  -reaction.ALCD2x, +reaction.EX_1hex_e,  -reaction.FRD2, -reaction.FRD3, +reaction.HX2COAR, +reaction.HXALDH,  -reaction.LDH_D, -reaction.PTA2, -reaction.PTAr |
| C8 | EX_iboh_e | glucose | anaerobic | 0,1388 | 8,7924 | +reaction.3MOBDC, -reaction.ACALD,  -reaction.ALCD2x, +reaction.EX_2mbtoh_e, +reaction.EX_2phetoh_e, +reaction.EX_iamoh_e, +reaction.EX_iboh_e, -reaction.FRD2,  -reaction.FRD3, +reaction.IBDH, +reaction.KARA1x, -reaction.LDH_D,  -reaction.OBTFL, -reaction.PFL, -reaction.PTA2,  -reaction.PTAr |
| D8 | EX_mal__L_e | glucose | anaerobic | 0,0798 | 0 | -reaction.ACALD, -reaction.ACKr,  -reaction.ALCD2x, -reaction.DTARTD,  -reaction.FRD2, -reaction.FRD3, -reaction.FUM,  -reaction.LDH_D, -reaction.ME2, -reaction.MGSA,  -reaction.OBTFL, -reaction.PFL, -reaction.POX |
| E8 | EX_lac__D_e | glucose | microaerobic | 0,0846 | 0 | -reaction.ACALD, -reaction.ACKr,  -reaction.ALCD2x, -reaction.FRD2, -reaction.FRD3,  -reaction.LDH_D, -reaction.LDH_D2,  -reaction.OBTFL, -reaction.PFL, -reaction.POX,  -reaction.PPS, -reaction.PTA2, -reaction.PTAr |
| F8 | EX_3hb_co_la_e | glucose | microaerobic | 0,1879 | 0 | +reaction.3HB_LA_POLYM, -reaction.ACALD,  -reaction.ALCD2x, +reaction.EX_3hb_co_la_e,  -reaction.FRD2, -reaction.FRD3, -reaction.OBTFL,  -reaction.PFL, +reaction.PHPB |
| G8 | EX_lac__D_e | glycerol | microaerobic | 0 | 0 | -reaction.ACALD, -reaction.ACKr,  -reaction.ALCD2x, -reaction.FRD2, -reaction.FRD3, -reaction.LDH_D, -reaction.LDH_D2,  -reaction.OBTFL, -reaction.PFL, -reaction.POX,  -reaction.PPS, -reaction.PTA2, -reaction.PTAr |
| H8 | EX_etoh_e | glucose | microaerobic | 0,1893 | 17,204 | -reaction.ACKr, -reaction.FRD2, -reaction.FRD3,  -reaction.LDH_D, -reaction.OBTFL, -reaction.PFL |
| I8 | EX_etoh_e | xylose | microaerobic | 0,1238 | 14,839 | -reaction.ACKr, -reaction.FRD2, -reaction.FRD3,  -reaction.LDH_D, -reaction.OBTFL, -reaction.PFL |
| A9 | None | glucose | microaerobic | 0 | 0 | -reaction.ACALD, -reaction.ALCD2x, +reaction.EX_2ptone_e, -reaction.LDH_D, +reaction.MKS, -reaction.POX, -reaction.PTA2,  -reaction.PTAr, +reaction.THE |
| B9 | EX_1boh_e | glucose+LB | anaerobic | 0,1039 | 5,7324 | +reaction.1BDH, -reaction.ACALD, -reaction.ACKr, -reaction.ALCD2x, +reaction.B2COAR, +reaction.BTALDH, +reaction.EX_1boh_e,  -reaction.FRD2, -reaction.FRD3, -reaction.LDH_D,  -reaction.MGSA, -reaction.OBTFL, -reaction.PFL |
| C9 | EX_lac__D_e | sucrose | anaerobic | 0 | 0 | -reaction.ACALD, -reaction.ALCD2x,  -reaction.FRD2, -reaction.FRD3, -reaction.GCALDD, -reaction.LCADi, -reaction.OBTFL, -reaction.PFL, -reaction.PTA2,  -reaction.PTAr |
| D9 | EX_3hb_e | glucose | anaerobic | 0,2415 | 0 | +reaction.3HB_POLYM, +reaction.EX_3hb_e,  -reaction.FDH4pp, -reaction.FDH5pp,  -reaction.FRD2, -reaction.FRD3, -reaction.HYD1pp, -reaction.HYD2pp, -reaction.HYD3pp,  -reaction.LDH_D, -reaction.PDH, +reaction.PHPB |
| E9 | EX_xylt_e | glucose+xylose | anaerobic | 0,1259 | 0 | -reaction.ACALD, -reaction.ACGAptspp,  -reaction.ACKr, -reaction.ALCD2x, +reaction.EX_xylt_e, -reaction.FORt2pp,  -reaction.FORtppi, -reaction.FRD2, -reaction.FRD3,  -reaction.GLCptspp, -reaction.LDH_D, +reaction.XYLR |
| F9 | EX_1poh_e | glucose+yeast extract | anaerobic | 0 | 0 | +reaction.1PDH, +reaction.2OBUTDC,  -reaction.ACALD, -reaction.ACHBS,  -reaction.ACLS, -reaction.ALCD2x, +reaction.BMALDH, +reaction.BMALHYD, +reaction.CIMHYD, +reaction.CIMSY, +reaction.EX_1poh_e, -reaction.FRD2,  -reaction.FRD3, -reaction.IPPS, -reaction.LDH_D,  -reaction.PTA2, -reaction.PTAr |
| G9 | EX_lac__L_e | glycerol | microaerobic | 0,0501 | 7,2256 | -reaction.ACALD, -reaction.ALCD2x,  -reaction.FRD2, -reaction.FRD3, -reaction.LDH_D, +reaction.LDH_L, -reaction.MGSA, -reaction.PTA2, -reaction.PTAr |
| H9 | EX_1boh_e | glucose | anaerobic | 0,1972 | 8,225 | +reaction.1BDH, -reaction.ACACCT,  -reaction.ACALD, -reaction.ALCD2x, +reaction.B2COAR, +reaction.BTALDH,  -reaction.BUTCT, -reaction.DHACOAH, +reaction.EX_1boh_e, -reaction.FRD2,  -reaction.FRD3, -reaction.HADPCOADH3,  -reaction.HXCT, -reaction.LDH_D,  -reaction.OXDHCOAT, -reaction.PTA2,  -reaction.PTAr, -reaction.REPHACCOAI |
| I9 | EX_succ_e | glucose+LB | anaerobic | 0,105 | 0,0348 | -reaction.LDH_D, -reaction.OBTFL, -reaction.PFL, +reaction.PYC |
| A10 | EX_but_e | glucose | anaerobic | 0,1639 | 0 | -reaction.ACACCT, -reaction.ACALD,  -reaction.ALCD2x, +reaction.B2COAR,  -reaction.BUTCT, +reaction.BUTTH,  -reaction.DHACOAH, -reaction.FRD2,  -reaction.FRD3, -reaction.HADPCOADH3,  -reaction.HXCT, -reaction.LDH_D,  -reaction.OXDHCOAT, -reaction.PTA2,  -reaction.PTAr, -reaction.REPHACCOAI |
| B10 | EX_h2_e | glycerol | anaerobic | 0,0868 | 17,3145 | -reaction.FDH4pp, -reaction.FDH5pp,  -reaction.FRD2, -reaction.FRD3, -reaction.LDH_D,  -reaction.MGSA, -reaction.NO3R1pp,  -reaction.NO3R2pp, -reaction.PPC |
| C10 | EX_but_e | glucose | anaerobic | 0,1659 | 0 | -reaction.ACALD, -reaction.ACGAptspp,  -reaction.ALCD2x, +reaction.B2COAR, +reaction.BUTTH, -reaction.FRD2, -reaction.FRD3,  -reaction.GLCptspp, -reaction.LDH_D,  -reaction.POX |
| D10 | EX_but_e | glucose+LB | anaerobic | 0,0918 | 0 | -reaction.ACALD, -reaction.ACKr,  -reaction.ALCD2x, +reaction.B2COAR, +reaction.BUTTH, -reaction.FRD2, -reaction.FRD3,  -reaction.LDH_D, -reaction.PTA2, -reaction.PTAr |
| E10 | EX_lac__L_e | mannitol | microaerobic | 0,1082 | 0 | -reaction.ACALD, -reaction.ALCD2x,  -reaction.FRD2, -reaction.FRD3, -reaction.LDH_D, +reaction.LDH_L, -reaction.L_LACD2,  -reaction.L_LACD3, -reaction.MGSA,  -reaction.PTA2, -reaction.PTAr |
| F10 | EX_1boh_e | glucose+yeast extract | anaerobic | 0 | 0 | +reaction.1BDH, -reaction.ACALD,  -reaction.ACOLIPAabctex, -reaction.ALCD2x, +reaction.B2COAR, +reaction.BTALDH,  -reaction.CLIPAabctex, -reaction.COLIPAPabctex,  -reaction.COLIPAabctex,  -reaction.ECA4COLIPAabctex,  -reaction.ENLIPAabctex, +reaction.EX_1boh_e,  -reaction.FRD2, -reaction.FRD3,  -reaction.K2L4Aabctex, -reaction.LDH_D,  -reaction.LIPAabctex,  -reaction.O16A4COLIPAabctex, -reaction.PTA2,  -reaction.PTAr |
| G10 | EX_crot_e | glycerol | anaerobic | 0,0387 | 0 | -reaction.ACALD, -reaction.ALCD2x, +reaction.CROT, +reaction.EX_crot_e,  -reaction.FRD2, -reaction.FRD3, -reaction.LDH_D,  -reaction.POX, -reaction.PTA2, -reaction.PTAr |
| H10 | EX_xylt_e | glucose+xylose | anaerobic | 0,0538 | 0 | -reaction.ACALD, -reaction.ACGAptspp,  -reaction.ACKr, -reaction.ALCD2x,  -reaction.DXYLK, +reaction.EX_xylt_e,  -reaction.FORt2pp, -reaction.FORtppi,  -reaction.FRD2, -reaction.FRD3,  -reaction.GLCptspp, -reaction.LDH_D,  -reaction.XYLI1, -reaction.XYLI2, -reaction.XYLK, +reaction.XYLR |

Table S5 Predicted metabolite targets and correspondent equivalent reaction knockout targets identified by MARSI for strain designs that have been experimentally validated and reproduced in silico.

| Key | Base design | Replaced targets | Metabolite target | Old fitness | Fitness |
| --- | --- | --- | --- | --- | --- |
| B1 | -reaction.ACALD, -reaction.ALCD2x, -reaction.PTA2 | -reaction.PTAr | ☣actp | 0,3285 | 0,3285 |
| D2 | -reaction.FRD3, -reaction.LDH_D, -reaction.OBTFL, -reaction.PFL,  -reaction.ACALD, -reaction.ACKr, -reaction.ALCD2x, -reaction.FORt2pp,  -reaction.FORtppi, +reaction.LDH_L | -reaction.FRD2 | ☣succ | 0,3143 | 0,3102 |
| D2 | -reaction.OBTFL, -reaction.PFL, -reaction.ACALD, -reaction.ACKr,  -reaction.ALCD2x, -reaction.FORt2pp, -reaction.FORtppi, -reaction.FRD2,  -reaction.FRD3, +reaction.LDH_L | -reaction.LDH_D | ☣lac__D | 0,3143 | 0,3143 |
| D2 | -reaction.ACALD, -reaction.ACKr, -reaction.ALCD2x, -reaction.FORt2pp,  -reaction.FORtppi, -reaction.FRD2, -reaction.FRD3, -reaction.LDH_D,  -reaction.OBTFL, +reaction.LDH_L | -reaction.PFL | ☣for | 0,3143 | 0,3285 |
| E2 | -reaction.FRD3, -reaction.OBTFL, -reaction.PFL, -reaction.ACALD,  -reaction.ACKr, -reaction.ALCD2x, -reaction.FORt2pp, -reaction.FORtppi | -reaction.FRD2 | ☣succ | 0,3143 | 0,3102 |
| E2 | -reaction.ACALD, -reaction.ACKr, -reaction.ALCD2x, -reaction.FORt2pp,  -reaction.FORtppi, -reaction.FRD2, -reaction.FRD3, -reaction.OBTFL | -reaction.PFL | ☣for | 0,3143 | 0,3285 |
| F2 | -reaction.AKGDH, -reaction.ALCD2x, -reaction.ATPS4rpp,  -reaction.FORt2pp, -reaction.FORtppi, -reaction.FRD2, -reaction.FRD3,  -reaction.LDH_D, -reaction.OBTFL, -reaction.PFL | -reaction.ACALD | ☣acald | 0,5124 | 0,5124 |
| F2 | -reaction.ATPS4rpp, -reaction.FORt2pp, -reaction.FORtppi, -reaction.FRD2,  -reaction.FRD3, -reaction.LDH_D, -reaction.OBTFL, -reaction.PFL,  -reaction.ACALD, -reaction.AKGDH | -reaction.ALCD2x | ☣etoh | 0,5124 | 0,5124 |
| F2 | -reaction.LDH_D, -reaction.OBTFL, -reaction.PFL, -reaction.ACALD,  -reaction.AKGDH, -reaction.ALCD2x, -reaction.ATPS4rpp,  -reaction.FORt2pp, -reaction.FORtppi, -reaction.FRD2 | -reaction.FRD3 | ☣succ | 0,5124 | 0,5124 |
| H2 | -reaction.ACALD, -reaction.ALCD2x, -reaction.PTA2 | -reaction.PTAr | ☣actp | 0,3285 | 0,3285 |
| A3 | -reaction.ACALD, -reaction.ALCD2x, -reaction.HEX1, -reaction.PFK,  -reaction.PFK_2, -reaction.PFK_3, -reaction.PTA2 | -reaction.PTAr | ☣actp | 0,3230 | 0,3230 |
| H3 | -reaction.FRD3, -reaction.OBTFL, -reaction.PFL, -reaction.ACALD,  -reaction.ACKr, -reaction.ALCD2x | -reaction.FRD2 | ☣succ | 0,3143 | 0,3102 |
| I3 | -reaction.FRD2, -reaction.FRD3, -reaction.LDH_D, -reaction.MGSA,  -reaction.OBTFL, -reaction.PFL, -reaction.ACALD, -reaction.ACKr, +reaction.LDH_L | -reaction.ALCD2x | ☣acald | 0,3225 | 0,3225 |
| I3 | -reaction.FRD2, -reaction.FRD3, -reaction.LDH_D, -reaction.MGSA,  -reaction.OBTFL, -reaction.PFL, -reaction.ACALD, -reaction.ACKr, +reaction.LDH_L | -reaction.ALCD2x | ☣acald, ☣etoh | 0,3225 | 0,3225 |
| I3 | -reaction.FRD3, -reaction.LDH_D, -reaction.MGSA, -reaction.OBTFL,  -reaction.PFL, -reaction.ACALD, -reaction.ACKr, -reaction.ALCD2x, +reaction.LDH_L | -reaction.FRD2 | ☣succ | 0,3225 | 0,3102 |
| I3 | -reaction.MGSA, -reaction.OBTFL, -reaction.PFL, -reaction.ACALD,  -reaction.ACKr, -reaction.ALCD2x, -reaction.FRD2, -reaction.FRD3, +reaction.LDH_L | -reaction.LDH_D | ☣lac__D | 0,3225 | 0,3225 |
| A4 | -reaction.FRD3, -reaction.OBTFL, -reaction.PFL, -reaction.ACALD,  -reaction.ACKr, -reaction.ALCD2x | -reaction.FRD2 | ☣succ | 0,3143 | 0,3102 |
| B4 | -reaction.FRD2, -reaction.FRD3, -reaction.MGSA, -reaction.OBTFL,  -reaction.PFL, -reaction.ACALD, -reaction.ACKr | -reaction.ALCD2x | ☣acald | 0,3225 | 0,3225 |
| B4 | -reaction.FRD2, -reaction.FRD3, -reaction.MGSA, -reaction.OBTFL,  -reaction.PFL, -reaction.ACALD, -reaction.ACKr | -reaction.ALCD2x | ☣acald, ☣etoh | 0,3225 | 0,3225 |
| B4 | -reaction.FRD3, -reaction.MGSA, -reaction.OBTFL, -reaction.PFL,  -reaction.ACALD, -reaction.ACKr, -reaction.ALCD2x | -reaction.FRD2 | ☣succ | 0,3225 | 0,3102 |
| E4 | -reaction.FRD3, -reaction.HYD1pp, -reaction.HYD2pp, -reaction.HYD3pp,  -reaction.LDH_D, -reaction.PDH, -reaction.FDH4pp, -reaction.FDH5pp, +reaction.PDC | -reaction.FRD2 | ☣succ | 0,4825 | 0,4828 |
| F4 | -reaction.FRD3, -reaction.OBTFL, -reaction.PDH, -reaction.PFL,  -reaction.POX, -reaction.PPS | -reaction.FRD2 | ☣succ | 0,3250 | 0,3253 |
| G4 | -reaction.FRD3, -reaction.PTA2, -reaction.PTAr | -reaction.FRD2 | ☣succ | 0,0039 | 0,0039 |
| B5 | -reaction.FRD3, -reaction.PTA2, -reaction.PTAr, -reaction.FHL | -reaction.FRD2 | ☣succ | 0,0039 | 0,0039 |
| C5 | -reaction.FRD3, -reaction.FRUpts2pp, -reaction.G6PDH2r,  -reaction.GAMptspp, -reaction.GLCptspp, -reaction.HEX1, -reaction.LDH_D, -reaction.MANptspp, -reaction.ME2, -reaction.NADH10, -reaction.NADH5,  -reaction.NADH9, -reaction.POX, -reaction.PTA2, -reaction.PTAr,  -reaction.ACGAptspp, -reaction.ACMANAptspp | -reaction.FRD2 | ☣succ | 0,0425 | 0,0426 |
| C5 | -reaction.MANptspp, -reaction.ME2, -reaction.NADH10, -reaction.NADH5,  -reaction.NADH9, -reaction.POX, -reaction.PTA2, -reaction.PTAr,  -reaction.ACGAptspp, -reaction.ACMANAptspp, -reaction.FRD2,  -reaction.FRD3, -reaction.FRUpts2pp, -reaction.G6PDH2r,  -reaction.GAMptspp, -reaction.GLCptspp, -reaction.HEX1 | -reaction.LDH_D | ☣lac__D | 0,0425 | 0,0425 |
| F5 | -reaction.FRD3, -reaction.LDH_D, -reaction.PTA2, -reaction.PTAr,  -reaction.ACALD, -reaction.ALCD2x, +reaction.1BDH, +reaction.B2COAR, +reaction.BTALDH, +reaction.EX_1boh_e | -reaction.FRD2 | ☣succ | 0,1622 | 0,1624 |
| F5 | -reaction.PTA2, -reaction.PTAr, -reaction.ACALD, -reaction.ALCD2x,  -reaction.FRD2, -reaction.FRD3, +reaction.1BDH, +reaction.B2COAR, +reaction.BTALDH, +reaction.EX_1boh_e | -reaction.LDH_D | ☣lac__D | 0,1622 | 0,1622 |
| F5 | -reaction.ACALD, -reaction.ALCD2x, -reaction.FRD2, -reaction.FRD3,  -reaction.LDH_D, -reaction.PTA2, +reaction.1BDH, +reaction.B2COAR, +reaction.BTALDH, +reaction.EX_1boh_e | -reaction.PTAr | ☣actp | 0,1622 | 0,1622 |
| I5 | -reaction.MGSA, -reaction.OBTFL, -reaction.PFL, -reaction.POX,  -reaction.ACALD, -reaction.ACKr, -reaction.ALCD2x, -reaction.FORt2pp,  -reaction.FORtppi | -reaction.LDH_D | ☣lac__D | 0,0830 | 0,0830 |
| A6 | -reaction.FRD3, -reaction.LDH_D, -reaction.OBTFL, -reaction.PFL,  -reaction.PTA2, -reaction.PTAr, -reaction.ACALD, -reaction.ALCD2x, +reaction.ACLDC, +reaction.EX_btd__RR_e, +reaction.sADHx, +reaction.sADHy | -reaction.FRD2 | ☣succ | 0,0244 | 0,0279 |
| A6 | -reaction.OBTFL, -reaction.PFL, -reaction.PTA2, -reaction.PTAr,  -reaction.ACALD, -reaction.ALCD2x, -reaction.FRD2, -reaction.FRD3, +reaction.ACLDC, +reaction.EX_btd__RR_e, +reaction.sADHx, +reaction.sADHy | -reaction.LDH_D | ☣lac__D | 0,0244 | 0,0244 |
| B6 | -reaction.FRD3, -reaction.OBTFL, -reaction.PFL, -reaction.ACALD,  -reaction.ALCD2x | -reaction.FRD2 | ☣succ | 0,3143 | 0,3102 |
| C6 | -reaction.FRD3, -reaction.LDH_D, -reaction.OBTFL, -reaction.PFL,  -reaction.PTA2, -reaction.PTAr, -reaction.ACALD, -reaction.ALCD2x, +reaction.ACLDC, +reaction.EX_btd__meso_e, +reaction.sADHx | -reaction.FRD2 | ☣succ | 0,0244 | 0,0279 |
| C6 | -reaction.OBTFL, -reaction.PFL, -reaction.PTA2, -reaction.PTAr,  -reaction.ACALD, -reaction.ALCD2x, -reaction.FRD2, -reaction.FRD3, +reaction.ACLDC, +reaction.EX_btd__meso_e, +reaction.sADHx | -reaction.LDH_D | ☣lac__D | 0,0244 | 0,0244 |
| F6 | -reaction.FRD3 | -reaction.FRD2 | ☣succ | 0,2139 | 0,2141 |
| H6 | -reaction.ALCD2x, -reaction.PPC, -reaction.ACALD | -reaction.ACKr | ☣ac | 0,1277 | 0,1277 |
| H6 | -reaction.ALCD2x, -reaction.PPC, -reaction.ACALD | -reaction.ACKr | ☣ac, ☣actp | 0,1277 | 0,1277 |
| B7 | -reaction.FRD3, -reaction.LDH_D, -reaction.OBTFL, -reaction.PFL,  -reaction.PTA2, -reaction.PTAr, -reaction.ACALD, -reaction.ALCD2x, +reaction.3MOBDC, +reaction.EX_2mbtoh_e, +reaction.EX_2phetoh_e, +reaction.EX_iamoh_e, +reaction.EX_iboh_e, +reaction.IBDH | -reaction.FRD2 | ☣succ | 0,0420 | 0,0418 |
| B7 | -reaction.OBTFL, -reaction.PFL, -reaction.PTA2, -reaction.PTAr,  -reaction.ACALD, -reaction.ALCD2x, -reaction.FRD2, -reaction.FRD3, +reaction.3MOBDC, +reaction.EX_2mbtoh_e, +reaction.EX_2phetoh_e, +reaction.EX_iamoh_e, +reaction.EX_iboh_e, +reaction.IBDH | -reaction.LDH_D | ☣lac__D | 0,0420 | 0,0420 |
| E7 | -reaction.MDH, -reaction.OBTFL, -reaction.PFL, -reaction.ACALD,  -reaction.ALCD2x, +reaction.4HBACT, +reaction.4HBTALDDH, +reaction.AKGDC, +reaction.BTDP2, +reaction.EX_14btd_e, +reaction.EX_4hdxbld_e, +reaction.EX_gbl_e, +reaction.GBL_PROD, +reaction.SUCCALDH | -reaction.LDH_D | ☣lac__D | 0,0904 | 0,0904 |
| G7 | -reaction.FRD3, -reaction.LDH_D, -reaction.PTA2, -reaction.PTAr,  -reaction.ACALD, -reaction.ALCD2x, +reaction.1BDH, +reaction.B2COAR, +reaction.BTALDH, +reaction.EX_1boh_e | -reaction.FRD2 | ☣succ | 0,0554 | 0,0555 |
| G7 | -reaction.ACALD, -reaction.ALCD2x, -reaction.FRD2, -reaction.FRD3,  -reaction.LDH_D, -reaction.PTA2, +reaction.1BDH, +reaction.B2COAR, +reaction.BTALDH, +reaction.EX_1boh_e | -reaction.PTAr | ☣actp | 0,0554 | 0,0554 |
| H7 | -reaction.FRD3, -reaction.PTA2, -reaction.PTAr, -reaction.ACALD,  -reaction.ALCD2x, +reaction.1BDH, +reaction.B2COAR, +reaction.BTALDH, +reaction.EX_1boh_e | -reaction.FRD2 | ☣succ | 0,0239 | 0,0239 |
| I7 | -reaction.OBTFL, -reaction.PFL, -reaction.ACALD, -reaction.ACGAptspp,  -reaction.ALCD2x, -reaction.GLCptspp | -reaction.LDH_D | ☣lac__D | 0,0255 | 0,0255 |
| A8 | -reaction.FRD3, -reaction.OBTFL, -reaction.PFL, -reaction.ACALD,  -reaction.ALCD2x | -reaction.FRD2 | ☣succ | 0,3144 | 0,3103 |
| B8 | -reaction.FRD3, -reaction.LDH_D, -reaction.PTA2, -reaction.PTAr,  -reaction.ACALD, -reaction.ALCD2x, +reaction.1HDH, +reaction.EX_1hex_e, +reaction.HX2COAR, +reaction.HXALDH | -reaction.FRD2 | ☣succ | 0,0370 | 0,0370 |
| B8 | -reaction.ACALD, -reaction.ALCD2x, -reaction.FRD2, -reaction.FRD3,  -reaction.LDH_D, -reaction.PTA2, +reaction.1HDH, +reaction.EX_1hex_e, +reaction.HX2COAR, +reaction.HXALDH | -reaction.PTAr | ☣actp | 0,0370 | 0,0370 |
| C8 | -reaction.OBTFL, -reaction.PFL, -reaction.PTA2, -reaction.PTAr,  -reaction.ACALD, -reaction.ALCD2x, -reaction.FRD2, -reaction.FRD3, +reaction.3MOBDC, +reaction.EX_2mbtoh_e, +reaction.EX_2phetoh_e, +reaction.EX_iamoh_e, +reaction.EX_iboh_e, +reaction.IBDH, +reaction.KARA1x | -reaction.LDH_D | ☣lac__D | 0,1221 | 0,1221 |
| H8 | -reaction.OBTFL, -reaction.PFL, -reaction.ACKr, -reaction.FRD2,  -reaction.FRD3 | -reaction.LDH_D | ☣lac__D | 0,3256 | 0,3256 |
| I8 | -reaction.OBTFL, -reaction.PFL, -reaction.ACKr, -reaction.FRD2,  -reaction.FRD3 | -reaction.LDH_D | ☣lac__D | 0,1837 | 0,1837 |
| B9 | -reaction.FRD2, -reaction.FRD3, -reaction.LDH_D, -reaction.MGSA,  -reaction.OBTFL, -reaction.PFL, -reaction.ACALD, -reaction.ACKr, +reaction.1BDH, +reaction.B2COAR, +reaction.BTALDH, +reaction.EX_1boh_e | -reaction.ALCD2x | ☣acald | 0,0595 | 0,0595 |
| B9 | -reaction.FRD2, -reaction.FRD3, -reaction.LDH_D, -reaction.MGSA,  -reaction.OBTFL, -reaction.PFL, -reaction.ACALD, -reaction.ACKr, +reaction.1BDH, +reaction.B2COAR, +reaction.BTALDH, +reaction.EX_1boh_e | -reaction.ALCD2x | ☣acald, ☣etoh | 0,0595 | 0,0595 |
| B9 | -reaction.FRD3, -reaction.LDH_D, -reaction.MGSA, -reaction.OBTFL,  -reaction.PFL, -reaction.ACALD, -reaction.ACKr, -reaction.ALCD2x, +reaction.1BDH, +reaction.B2COAR, +reaction.BTALDH, +reaction.EX_1boh_e | -reaction.FRD2 | ☣succ | 0,0595 | 0,0578 |
| B9 | -reaction.MGSA, -reaction.OBTFL, -reaction.PFL, -reaction.ACALD,  -reaction.ACKr, -reaction.ALCD2x, -reaction.FRD2, -reaction.FRD3, +reaction.1BDH, +reaction.B2COAR, +reaction.BTALDH, +reaction.EX_1boh_e | -reaction.LDH_D | ☣lac__D | 0,0595 | 0,0595 |
| G9 | -reaction.FRD2, -reaction.FRD3, -reaction.LDH_D, -reaction.MGSA,  -reaction.PTA2, -reaction.PTAr, -reaction.ACALD, +reaction.LDH_L | -reaction.ALCD2x | ☣acald | 0,0362 | 0,0362 |
| G9 | -reaction.FRD2, -reaction.FRD3, -reaction.LDH_D, -reaction.MGSA,  -reaction.PTA2, -reaction.PTAr, -reaction.ACALD, +reaction.LDH_L | -reaction.ALCD2x | ☣acald, ☣etoh | 0,0362 | 0,0362 |
| G9 | -reaction.FRD3, -reaction.LDH_D, -reaction.MGSA, -reaction.PTA2,  -reaction.PTAr, -reaction.ACALD, -reaction.ALCD2x, +reaction.LDH_L | -reaction.FRD2 | ☣succ | 0,0362 | 0,0362 |
| G9 | -reaction.MGSA, -reaction.PTA2, -reaction.PTAr, -reaction.ACALD,  -reaction.ALCD2x, -reaction.FRD2, -reaction.FRD3, +reaction.LDH_L | -reaction.LDH_D | ☣lac__D | 0,0362 | 0,0362 |
| G9 | -reaction.ACALD, -reaction.ALCD2x, -reaction.FRD2, -reaction.FRD3,  -reaction.LDH_D, -reaction.MGSA, -reaction.PTA2, +reaction.LDH_L | -reaction.PTAr | ☣actp | 0,0362 | 0,0362 |
| H9 | -reaction.FRD3, -reaction.HADPCOADH3, -reaction.HXCT,  -reaction.LDH_D, -reaction.OXDHCOAT, -reaction.PTA2, -reaction.PTAr,  -reaction.REPHACCOAI, -reaction.ACACCT, -reaction.ACALD,  -reaction.ALCD2x, -reaction.BUTCT, -reaction.DHACOAH, +reaction.1BDH, +reaction.B2COAR, +reaction.BTALDH, +reaction.EX_1boh_e | -reaction.FRD2 | ☣succ | 0,1622 | 0,1623 |
| H9 | -reaction.OXDHCOAT, -reaction.PTA2, -reaction.PTAr,  -reaction.REPHACCOAI, -reaction.ACACCT, -reaction.ACALD,  -reaction.ALCD2x, -reaction.BUTCT, -reaction.DHACOAH, -reaction.FRD2,  -reaction.FRD3, -reaction.HADPCOADH3, -reaction.HXCT, +reaction.1BDH, +reaction.B2COAR, +reaction.BTALDH, +reaction.EX_1boh_e | -reaction.LDH_D | ☣lac__D | 0,1622 | 0,1622 |
| H9 | -reaction.REPHACCOAI, -reaction.ACACCT, -reaction.ACALD,  -reaction.ALCD2x, -reaction.BUTCT, -reaction.DHACOAH, -reaction.FRD2,  -reaction.FRD3, -reaction.HADPCOADH3, -reaction.HXCT,  -reaction.LDH_D, -reaction.OXDHCOAT, -reaction.PTA2, +reaction.1BDH, +reaction.B2COAR, +reaction.BTALDH, +reaction.EX_1boh_e | -reaction.PTAr | ☣actp | 0,1622 | 0,1622 |
| B10 | -reaction.FRD3, -reaction.LDH_D, -reaction.MGSA, -reaction.NO3R1pp,  -reaction.NO3R2pp, -reaction.PPC, -reaction.FDH4pp, -reaction.FDH5pp | -reaction.FRD2 | ☣succ | 0,1503 | 0,1504 |

Table S6 Number of metabolite analogues retrieved from MARSI database.

|  | Metabolite Name | #Hits | #Hits (<50% Similarity) |
| --- | --- | --- | --- |
| *3hpppn_c* | 3-(3-hydroxy-phenyl)propionate | 6298 | 4129 |
| *23ddhb_c* | 2,3-Dihydro-2,3-dihydroxybenzoate | 2296 | 1519 |
| *3hddcoa_c* | (S)-3-Hydroxydodecanoyl-CoA | 6 | 0 |
| *2dr5p_c* | 2-Deoxy-D-ribose 5-phosphate | 226 | 155 |
| *35cgmp_c* | 3',5'-Cyclic GMP | 1254 | 428 |
| *2amsa_c* | 2-Aminomalonate semialdehyde | 1785 | 449 |
| *13dpg_c* | 3-Phospho-D-glyceroyl phosphate | 346 | 200 |
| *23dhba_c* | (2,3-Dihydroxybenzoyl)adenylate | 159 | 4 |
| *3ohcoa_c* | 3-Oxohexanoyl-CoA | 5 | 0 |
| *3dhgulnp_c* | 3-keto-L-gulonate-6-phosphate | 65 | 48 |
| *2dr1p_c* | 2-Deoxy-D-ribose 1-phosphate | 1149 | 508 |
| *2pg_c* | D-Glycerate 2-phosphate | 1056 | 472 |
| *15dap_c* | 1,5-Diaminopentane | 3181 | 925 |
| *2mcacn_c* | Cis-2-Methylaconitate | 902 | 566 |
| *12ppd__r_c* | (R)-Propane-1,2-diol | 244 | 193 |
| *2h3oppan_c* | 2-Hydroxy-3-oxopropanoate | 964 | 361 |
| *2dh3dgal_c* | 2-Dehydro-3-deoxy-D-galactonate | 1902 | 890 |
| *3hcinnm_c* | 3-hydroxycinnamic acid | 6608 | 4684 |
| *1ddecg3p_c* | 1-dodecanoyl-sn-glycerol 3-phosphate | 1308 | 41 |
| *3hhcoa_c* | (S)-3-Hydroxyhexanoyl-CoA | 4 | 0 |
| *2mcit_c* | 2-Methylcitrate | 1061 | 517 |
| *23doguln_c* | 2,3-Dioxo-L-gulonate | 1951 | 905 |
| *3oddcoa_c* | 3-Oxododecanoyl-CoA | 9 | 4 |
| *1tdecg3p_c* | 1-tetradecanoyl-sn-glycerol 3-phosphate | 918 | 26 |
| *3odcoa_c* | 3-Oxodecanoyl-CoA | 12 | 4 |
| *23dappa_c* | 2,3-diaminopropionate | 3025 | 851 |
| *12ppd__s_c* | (S)-Propane-1,2-diol | 1207 | 575 |
| *2ddg6p_c* | 2-Dehydro-3-deoxy-D-gluconate 6-phosphate | 102 | 73 |
| *2tpr3dpcoa_c* | 2'-(5''-triphosphoribosyl)-3'-dephospho-CoA | 10 | 1 |
| *3hadpcoa_c* | (3S)-3-Hydroxyadipyl-CoA | 2 | 0 |
| *3ohdcoa_c* | 3-Oxohexadecanoyl-CoA | 3 | 0 |
| *2aobut_c* | L-2-Amino-3-oxobutanoate | 2856 | 818 |
| *2shchc_c* | 2-Succinyl-6-hydroxy-2,4-cyclohexadiene-1-carboxylate | 319 | 182 |
| *2dh3dgal6p_c* | 2-Dehydro-3-deoxy-D-galactonate 6-phosphate | 102 | 73 |
| *2ddglcn_c* | 2-Dehydro-3-deoxy-D-gluconate | 1902 | 890 |
| *3hocoa_c* | (S)-3-Hydroxyoctanoyl-CoA | 6 | 1 |
| *3ohodcoa_c* | 3-Oxooctadecanoyl-CoA | 2 | 0 |
| *25dkglcn_c* | 2,5-diketo-D-gluconate | 1833 | 779 |
| *3hdcoa_c* | (S)-3-Hydroxydecanoyl-CoA | 12 | 2 |
| *2pglyc_c* | 2-Phosphoglycolate | 1121 | 564 |


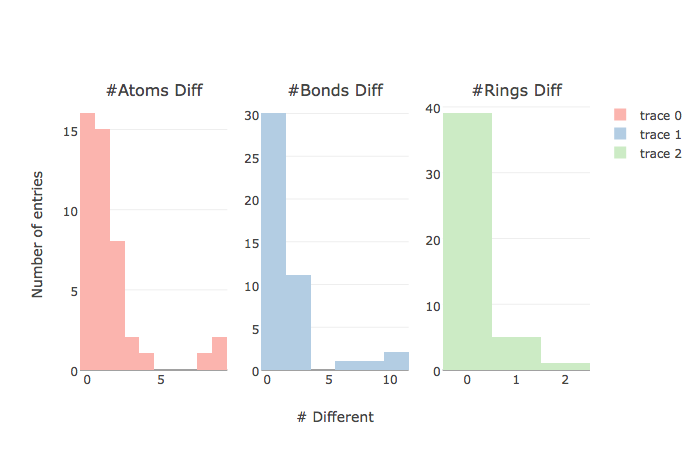


Figure S1 Number of metabolite analogues from our selected metabolite-antimetabolite pairs that can be retrieved using the difference between the number of atoms, the number of bonds and the number of rings using different cutoffs.


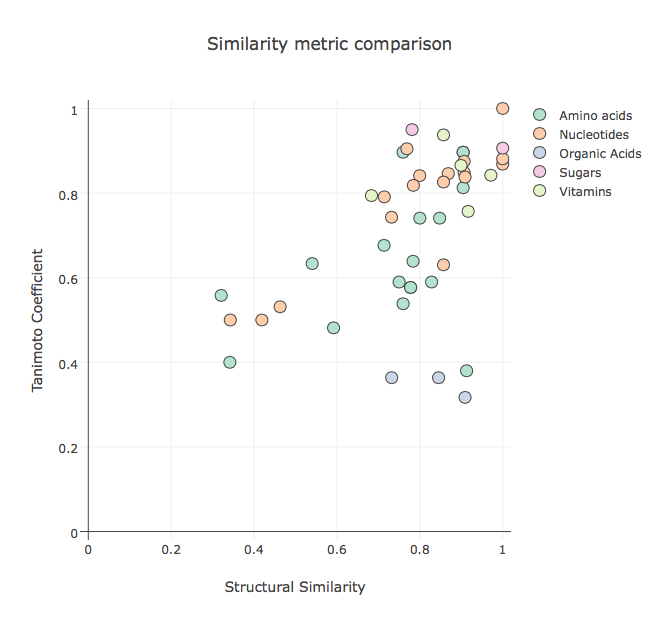


Figure S2 Comparison between Tanimoto coefficients and structural similarity. We can capture almost all known metabolite analogues with a similarity of 0.5.


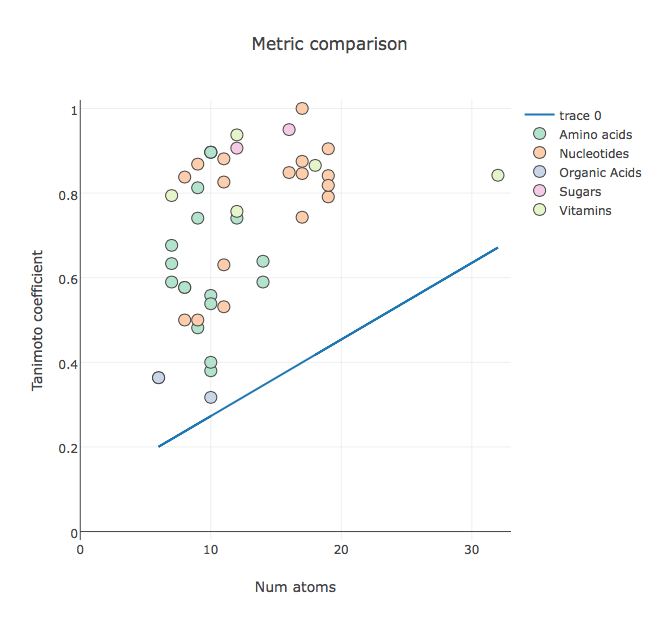


Figure S3 Tanimoto coefficient vs. number of atoms. The distribution of the number of atoms per molecule. The line represents the linear regression with adjusted intercept. This line was used to determine the cutoff value for smaller compounds.


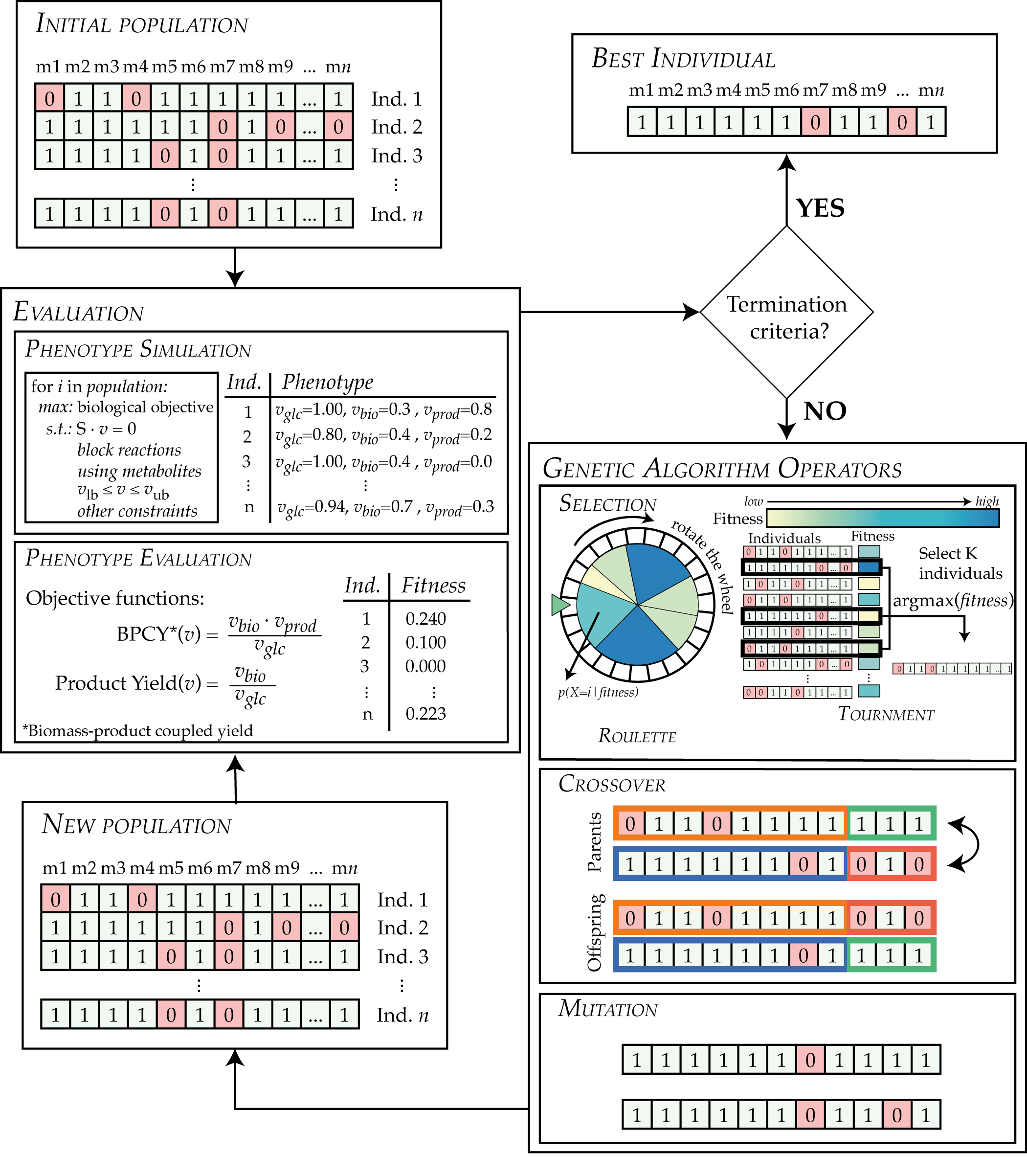


Figure S4 OptMet algorithm workflow. The algorithm starts with the generation of a population containing possible solutions. At each iteration the solutions are evaluated and redefined, inspired in the principles of Darwin evolution. When the termination criteria is met, the algorithm stops and returns the best individual. Each iteration the best solution is kept.
